# Malaria in Farmed Ungulates: an Exciting New System for Comparative Parasitology

**DOI:** 10.1128/mSphere.00161-18

**Published:** 2018-04-18

**Authors:** Susan L. Perkins

**Affiliations:** a Sackler Institute for Comparative Genomics and Division of Invertebrate Zoology, American Museum of Natural History, New York, New York, USA

**Keywords:** deer, hemosporidian, malaria, ungulate

## Abstract

A wide array of vertebrates can serve as the intermediate hosts to malaria parasites (Apicomplexa: Haemosporida), such as birds, lizards, and several groups of mammals, including primates, bats, rodents, and ungulates. The latter group of hosts has not been intensively studied since early descriptions of a small set of taxa were published, but new reports of these parasites in both expected and new hosts have recently been published. A new paper reports the presence of Plasmodium odocoilei in farmed white-tailed deer in Florida, particularly in animals less than 1 year old, and provides evidence that the parasites may contribute to mortality in fawns. That paper opens new opportunities to study the malaria parasite-mammal interface in North America.

## Abstract (precis)

A wide array of vertebrates can serve as the intermediate hosts to malaria parasites (Apicomplexa: Haemosporida), such as birds, lizards, and several groups of mammals, including primates, bats, rodents, and ungulates. The latter group of hosts has not been intensively studied since early descriptions of a small set of taxa were published, but new reports of these parasites in both expected and new hosts have recently been published.

## COMMENTARY

Humans are infected with five different species of malaria parasites, but in terms of the overall diversity of this group, we are relatively unimportant hosts. The more than 600 other described lineages of hemosporidians use nonhuman primates, rodents, and bats, as well as birds and lizards, as their vertebrate hosts. These diverse lineages show differing life cycles and frequently use blood-feeding dipterans other than mosquitoes as definitive hosts (1, 2). Early in 2016, a set of three papers was published, each reporting finding hemosporidian (“malaria”) parasites infecting ungulate hosts. This was, in some ways, not surprising, given that several species collected from other ungulate hosts had been described previously, but no additional work had been done on these parasites in over 30 years. One of these papers (3) detailed new instances of parasites that were morphologically consistent with Plasmodium odocoilei, a species described by Garnham and Kuttler in 1980 (4), from white-tailed deer. The type host had been splenectomized; thus, there was some doubt of the natural transmission of this parasite into deer hosts. A second paper (5) reported hemosporidians from two geographically disparate hosts: domestic goats in Africa and water buffalo in Vietnam and Thailand. Finally, the third paper (6) provided data on hemosporidian sequences from duiker that had been killed for bush meat in western Africa. Those studies were exciting to those interested in hemosporidian diversity and evolution, for they reconfirmed previous reports of the parasites but also provided several host and regional extensions to the first descriptions. More important, though, was that these new samples had been collected at a time and using a method that enabled obtaining genetic data from ungulate parasites for the first time. Although originally described as being part of the genus *Plasmodium*, preliminary molecular systematic analyses described in each of these three papers showed them to be polyphyletic with respect to other *Plasmodium* species and, at least in some of the phylogenies, most closely related to parasites in the genus *Polychromophilus*, known to infect insectivorous bats (3, 6).

A new paper by Guggisberg et al. (7) now expands the observations of deer malaria in the United States by providing evidence for a high prevalence of these parasites in farmed white-tailed deer in Florida. The authors followed 33 fawns through their first year of life and observed that over 20% of them acquired infections of the hemosporidians. Sequence data from the mitochondrial cytochrome *b* gene confirmed that these were the same parasites as had been sampled in other white-tailed deer and that there was at least some (low) level of genetic diversity present in the Florida populations. Interestingly, the authors also showed a correlation between infection with the hemosporidians and mortality of the deer, providing evidence for virulence, particularly with respect to young animals, similar to the patterns observed in the human parasite Plasmodium falciparum, with which most mortality occurs in infants. The authors also found a correspondence of hemosporidian infection with the presence of a viral pathogen; thus, comorbidity may be a partial explanation for the higher mortality in malaria-positive animals. Although the deer parasites are quite distantly related to P. falciparum, this convergence of levels of virulence in young animals may offer an opportunity to better understand factors underlying these specific host-parasite dynamics. For instance, studies have shown that changes from the fetal to the adult microbiome in mice and humans are linked to susceptibility to malaria infections (8) and that some ungulate species possess β-globin genes that can cause red blood cells to sickle (9), akin to the very well known trait that confers resistance to malaria in humans. All of this underscores the importance of continued sampling of wild vertebrates for parasites—even vertebrates that are literally in our backyards or on our farms.
